# Productivity enhancement in L-lysine fermentation using oxygen-enhanced bioreactor and oxygen vector

**DOI:** 10.3389/fbioe.2023.1181963

**Published:** 2023-05-03

**Authors:** Jinduo Wang, Shuo Wang, Siyu Zhao, Pengjie Sun, Zhen Zhang, Qingyang Xu

**Affiliations:** ^1^ National and Local United Engineering Lab of Metabolic Control Fermentation Technology, Tianjin University of Science and Technology, Tianjin, China; ^2^ College of Biotechnology, Tianjin University of Science and Technology, Tianjin, China

**Keywords:** L-lysine, oxygen-enhanced bioreactor, metabolic flux, fermentation engineering, oxygen vectors

## Abstract

**Introduction:** L-lysine is a bulk product. In industrial production using high-biomass fermentation, the high density of bacteria and the intensity of production require sufficient cellular respiratory metabolism for support. Conventional bioreactors often have difficulty meeting the oxygen supply conditions for this fermentation process, which is not conducive to improving the sugar-amino acid conversion rate. In this study, we designed and developed an oxygen-enhanced bioreactor to address this problem.

**Methods:** This bioreactor optimizes the aeration mix using an internal liquid flow guide and multiple propellers.

**Results:** Compared with a conventional bioreactor, it improved the k_L_a from 367.57 to 875.64 h^-1^, an increase of 238.22%. The results show that the oxygen supply capacity of the oxygen-enhanced bioreactor is better than that of the conventional bioreactor. Its oxygenating effect increased the dissolved oxygen in the middle and late stages of fermentation by an average of 20%. The increased viability of *Corynebacterium glutamicum* LS260 in the mid to late stages of growth resulted in a yield of 185.3 g/L of L-lysine, 74.57% conversion of lysine from glucose, and productivity of 2.57 g/L/h, an increase of 11.0%, 6.01%, and 8.2%, respectively, over a conventional bioreactor. Oxygen vectors can further improve the production performance of lysine strains by increasing the oxygen uptake capacity of microorganisms. We compared the effects of different oxygen vectors on the production of L-lysine from LS260 fermentation and concluded that n-dodecane was the most suitable. Bacterial growth was smoother under these conditions, with a 2.78% increase in bacterial volume, a 6.53% increase in lysine production, and a 5.83% increase in conversion. The different addition times of the oxygen vectors also affected the final yield and conversion, with the addition of oxygen vectors at 0 h, 8 h, 16 h, and 24 h of fermentation increasing the yield by 6.31%, 12.44%, 9.93%, and 7.39%, respectively, compared to fermentation without the addition of oxygen vectors. The conversion rates increased by 5.83%, 8.73%, 7.13%, and 6.13%, respectively. The best results were achieved by adding oxygen vehicles at the 8th hour of fermentation, with a lysine yield of 208.36 g/L and a conversion rate of 83.3%. In addition, n-dodecane significantly reduced the amount of foam produced during fermentation, which is beneficial for fermentation control and equipment.

**Conclusion:** The new oxygen-enhanced bioreactor improves oxygen transfer efficiency, and oxygen vectors enhance the ability of cells to take up oxygen, which effectively solves the problem of insufficient oxygen supply during lysine fermentation. This study provides a new bioreactor and production solution for lysine fermentation.

## 1 Introduction

L-lysine belongs to the aspartic acid group of amino acids. Its synthesis pathway is closely related to both EMP and TCA pathways. The density of bacteria during L-lysine fermentation is very high, and the performance limitations of traditional equipment often lead to insufficient dissolved oxygen content during the fermentation process, which to some extent weakens the advantages of the L-lysine metabolic pathway.

Lysine-producing *Corynebacterium glutamicum* bacteria are strictly aerobic (FEEDAP et al., 2020), owing to the aerobic nature of the strain and the high acid production efficiency of lysine, which therefore place more stringent requirements on the oxygen supply during fermentation. A key direction for solving this problem is to increase the oxygen supply capacity of bioreactors (Meng et al., 2022). Schirmer et al. (2021) analyzed the effects of impeller type, size, and number on bioreactor effectiveness, focusing on the advantages and disadvantages of different stirring types. The characteristics, process parameters, and internal components of the anaerobic fermentation equipment were investigated by Abdelgadir et al. (2014) A new type of highly efficient anaerobic fermentation plant was designed and obtained under optimum conditions for bacterial growth (Nikakhtari et al., 2014). In this study, an efficient oxygen-enhanced bioreactor with a liquid flow guide and combined stirring blades was designed to solve the problem of an insufficient oxygen supply.

Oxygen vectors have been introduced into the fermentation broth in recent years as a new liquid phase that is non-toxic to microorganisms and eliminates foam produced during fermentation (Galaction et al., 2004; Ciobanu et al., 2020). This liquid phase reduces the resistance to oxygen transfer from gas to liquid and has become a new trend in enhanced oxygen transfer technology (Liu and Wu, 2006; Quijano et al., 2010). This liquid phase does not cause toxicity or inhibit the growth of microorganisms, is immiscible with the fermentation broth, and has a higher capacity to dissolve oxygen than water. The addition of oxygen vectors such as paraffin, n-hexane, and n-dodecane increases the k_L_a (oxygen transfer coefficient) value of the same reaction system by up to 300% in varying degrees and also has the mixed effect of reducing the stirring power. Xia et al. used oxygen vectors to enhance antroquinonol yield during fermentation (Rols et al., 1990; Lee and Armiger, 1991; Menge et al., 2001; Chen et al., 2019). Enhancement of *ß*-galactosidase production by *Escherichia coli* using n-dodecane as an oxygen vector was described by Ciobanu et al. (2020). Wang et al. (2019) successfully increased the titer of diacetyl in the fermentation broth of *Bacillus* sp. by adding an oxygen vector to the fermentation broth. Azmi et al. (2013)used liquid paraffin to enhance the production of tyrosine phenol lyase by *Citrobacter freundii* MTCC 2424, while increasing rapid fuel cell growth. In this study, the effect of oxygen vectors on the fermentation production of L-lysine by *C. glutamicum* was analyzed to enhance the ability of bacteria to take up oxygen.

## 2 Materials and methods

### 2.1 Strains and culture

The strain used in this study was *C. glutamicum* LS260, which is conserved in the Metabolic Engineering Research Laboratory of Tianjin University of Science and Technology. The inoculum for L-lysine fermentation in a 5 L bioreactor underwent two bacterial activations. Bacterial activation was performed using a test tube slant medium and an eggplant bottle slant medium. The slant medium contained 2.5 g of agar powder, 5 g of yeast extract, 2.5 g of tryptone, 2 g of KH_2_PO_4_, 1 g of MgSO_4_ and 4 g of (NH_4_)_2_SO_4_ per liter, dissolved by heating. Each test tube was filled with 9 mL and the aubergine bottle slants were filled with 40 mL, and the samples were sterilized by autoclaving at 121°C for 20 min. Cells were obtained from glycerol-conserved tubes, inoculated onto two test tube slants, and incubated at 32°C for 12 h. The second activation was carried out using two aubergine bottle slants, and the inoculated cells were obtained from the first activated test tube slants. The cells were incubated at 32°C for 12 h.

### 2.2 L-lysine fermentation in a 5L bioreactor

Batch replenishment fermentation was carried out in a 5 L fermenter (Baoxing, Shanghai, China). Cells obtained from the aubergine flask culture described in Section 2.1 were used as the first-stage inoculum, eluted using 200 mL saline, and the eluted cells were transferred to a fermenter containing 1,8 L of seed medium containing 15 g of maize pulp, 5 g of yeast powder, 7 g of peptone, 4 g of KH_2_PO_4_, 15 g of (NH_4_)_2_SO_4_ and 1.5 g of MgSO_4_ per liter of seed medium. The incubation temperature of the seed solution in the fermentation tank was 34°C. The temperature was controlled using a combination of a cold-water jacket and a heating sump.

### 2.3 Quantification of biomass, glucose, and amino acids

Biomass was determined by measuring the absorbance of the cultures at 600 nm. Glucose levels in the cultures were determined using an SBA biosensor analyzer (SBA40E, Institute of Biology, Shandong Academy of Sciences, Jinan, China). For amino acids without UV absorption, L-lysine and by-products in the fermentation broth were determined by high-performance liquid chromatography using an Agilent C18 (15 mm * 4.6 mm, 3.5 μm) column with 2,4-dinitrofluorobenzene as derivatized and pre-column derivatization, a mobile phase of 50% acetonitrile, 4.1 g/L CH_3_COONa, column temperature of 33°C, flow rate of 1 mL/min, detection wavelength 360 nm.

### 2.4 Statistics and analysis

Unless otherwise noted, all experiments were performed in triplicate. Statistical significance was determined using one-way analysis of variance (ANOVA), followed by Dunnett’s multiple comparison test. Statistical significance was defined as *p* ≤ 0.05.

### 2.5 Types and properties of oxygen vectors

**FIGURE 1:**
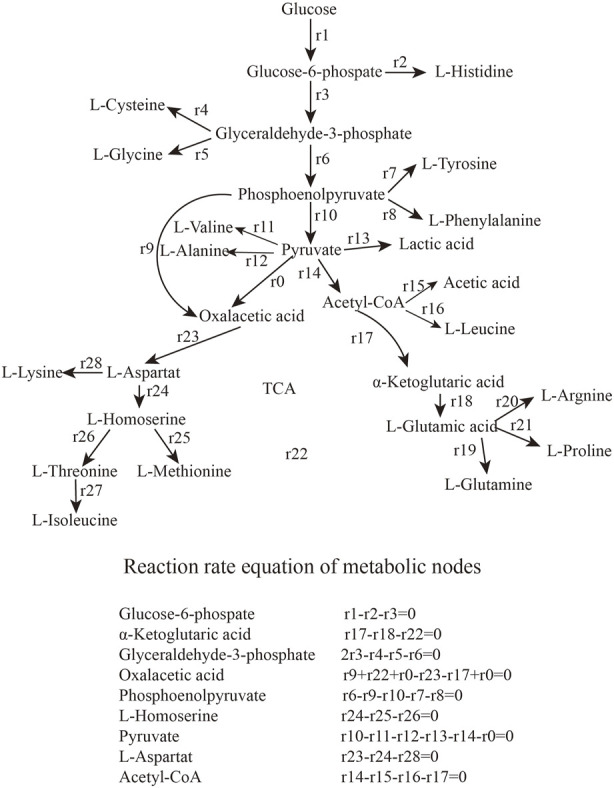
Metabolic networks and balancing equations.

**FIGURE 2:**
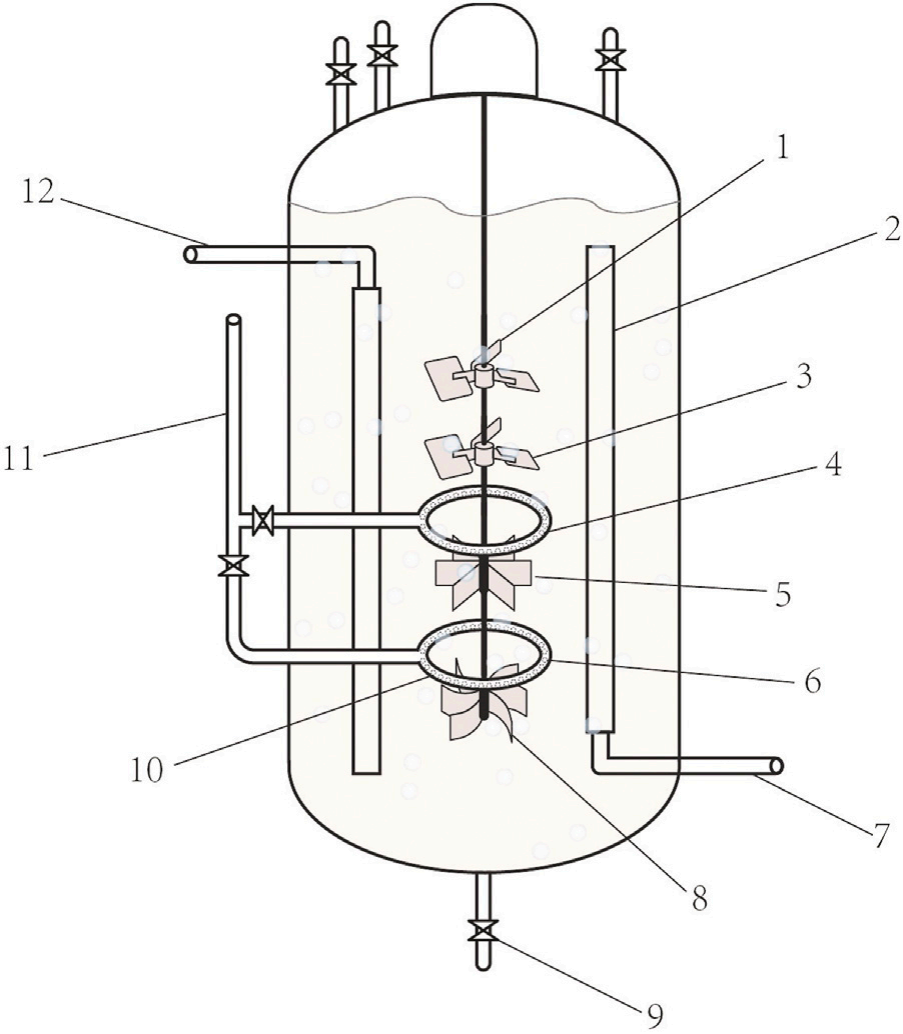
Oxygen-enhanced bioreactors. 1. Six inclined blade stirrers. 2. Liquid flow guides. 3. Six inclined blade stirrers 4. Air distributor. 5. Six straight-bladed stirrers, 6. Air distributor 4,7. Cooling water inlet,8. Six curved blade stirrer. 9. Export of the fermentation broth. 10. Air valves. 11. Air inlet. 12. Cooling water outlet.

The types and properties of the oxygen vectors are shown in Table 1, Figures 1, 2.

**TABLE 1:** Properties of oxygen vectors.

Type of oxygen vector	Surface tension (N·m^-1^·10^−3^)	Surface tension with water (N·m^-1^·10^−3^)	Capacity of dissolved oxygen (mg·L^-1^)	Spreading coefficient (N·m^-1^·10^−3^)	Density (kg·L ^-1^)
n-Dodecane	27.6	34.9	54.9	8.7	0.749
Perfluorocarbon	24.5	32.2	118.0	14.5	1.950
Liquid paraffin	24.7	51.4	39.5	−4.9	0.743
Water	71.2		6.3		

### 2.6 k_L_a calculation method

The K_L_a value was determined based on the equation:

$$dc/dt=K_{L}a\left(C^{*}-C\right)-\gamma$$

K_L_a is the volumetric dissolved oxygen coefficient (h^-1^), C* is the saturation dissolved oxygen value of the solution (mol/L), C is the dissolved oxygen concentration in the solution (mol/L), and γ is the oxygen consumption rate (mol/L/h).

In the presence of catalyst Co^2+^ or Cu^2+^ ions, sodium sulfite is rapidly oxidized to sodium sulfate. Taking advantage of this feature, we injected sodium sulfite into the reactor continuously at a certain rate under the condition of continuous gas stirring, and the oxygen consumption rate at this time can be calculated as follows.

γ=QM/2V

Q is the flow rate of Na_2_SO_3_ solution (L/h), M is the concentration of the solution (moL) with the addition of Na_2_SO_3_, and 2 indicates the consumption of 1 mol requires 2 mol Na_2_SO_3_ as the volume of liquid in the reactor (L). With the continuous addition of oxygen depleting agent, the dissolved oxygen in the reactor is consumed, and the dissolved oxygen value rises, thus forming the oxygen transfer force (C*-C) in the liquid phase of the reactor. In the steady state of continuous incubation, the dissolved oxygen concentration C in the reaction solution is constant and the dc/dt is zero. Therefore, K_L_a(C*-C) = *γ*, i.e., QM/2V = K_L_a(C*-C). The different values of K_L_a (i.e., different oxygen supply capacities) under different reactor operating conditions cause the dissolved oxygen concentration C to change accordingly. The concentration and flow rate of Na_2_SO_3_ solution in this process, as well as the volume of the reactor liquid, can be controlled. After measuring C/C* with the dissolved oxygen electrode, the K_L_a value can be easily calculated according to the following equation.

$$K_{L}a=QM/\left[2VC\left(1-\frac{C}{C^{*}}\right)\right]$$

### 2.7 Metabolic flux analysis

Metabolic flux analysis is an analytical method that specifically addresses the flux of metabolic pathways (Campit and Chandrasekaran, 2020; Antoniewicz, 2021). The quasi-steady-state assumption is that, with rapid intracellular metabolic reactions, none of the intracellular metabolites accumulate and are in a steady-state state with a change in concentration of 0 (Reimers and Reimers, 2016). The flux distribution for various intracellular biochemical reactions can also be calculated from multivariate equations after extracellular substances have been measured. This results in a metabolic flux map containing almost all relevant reactions and metabolites at steady-state (Razmilic et al., 2018). Metabolic flow distribution can be analyzed under different conditions and in different mutant strains (Soh et al., 2012).

## 3 Results and discussion

### 3.1 Multi-stirred paddle-linked oxygen-enhanced bioreactors

**FIGURE 3:**
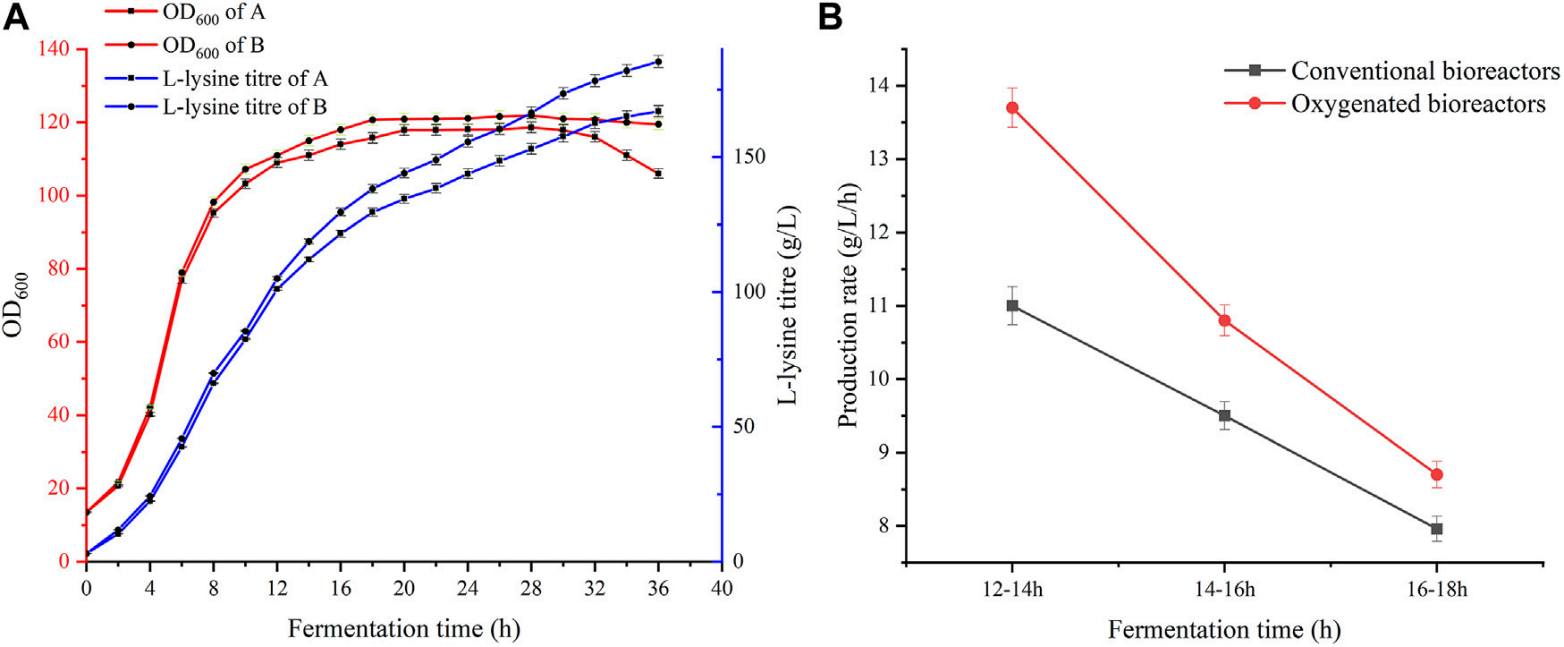
Comparison of fermentation in the two bioreactors. **(A)** Comparison of yield and biomass of the two bioreactors. **(B)** Comparison of productivity in the two bioreactors where fermentation was carried out for 12–18 h.

In the amino acid fermentation industry, most amino acid-producing bacteria are strictly aerobically fermented (Yamamoto et al., 2011). Fermentation results in a high density of bacteria and requires an extremely high oxygen supply (Okino et al., 2008). This problem is even more pronounced in bulk amino acids (L-glutamic acid, L-lysine, etc.), where very high k_L_a is often required to properly support fermentation, which places more stringent requirements on the oxygen supply to the equipment (Garcia-Ochoa and Gomez, 2005; Okino et al., 2005). In this study, a new type of oxygen-enhanced fermentation equipment was designed to maximize the efficiency of the oxygen supply to the equipment. The equipment combines the advantages of mechanically stirred and air-lift bioreactors (Aragão et al., 2020). We installed a liquid flow guide inside the conventional bioreactor, which solves the problem of the chaotic flow of the fermentation broth inside the tank, and tends to form dead ends for agitation (Farkya et al., 2004; Gill et al., 2008). Four sets of stirring fan blades were set up in the inflow tube. The order of the stirring fan blades from top to bottom was as follows: screw paddle, screw paddle, six straight blade stirring paddles, and six curved blade stirring paddles (the number of fan blades can be adjusted according to equipment size and liquid viscosity). The rapid downward flow of fermentation fluid occurred under the thrust of two sets of propellers and two screw sets. The liquid drove the gas downward through the six straight-blade stirring paddles, which efficiently broke up air bubbles. Smaller bubbles have a larger specific surface area, which facilitates the gas-liquid mass transfer. An air distributor and six curved blade turbines were located at the bottom, which further crushed the air bubbles. The interior of the liquid flow guide was hollow and contained a water inlet and outlet. This allowed it to be cooled internally by circulating water and allowed for precise temperature control. Two sets of air distributors were connected to the air inlets running through the side of the tank and were each provided with a control valve. As the six straight-bladed stirring paddles were more powerful and crushed the fluid better than the six curved-bladed stirring paddles, the airflow can be controlled by adjusting the air valve and aeration volume. When the stirrer speed was high, the upper and lower air supply valves could be opened to achieve a dual-pipeline air supply. When the swing mixing speed was low, only the lower air supply valve should be opened. The sum of the diameters of the exhaust holes was equal to the cross-sectional area of the exhaust duct. After the gas entered the guide tube, it dissolved in the downward flowing fermentation broth at high speed, increasing the flow distance of the gas in the fermentation broth, and then is crushed by six straight-bladed stirring paddles and six curved-bladed stirring paddles twice, thus increasing the dissolved oxygen rate of the fermentation broth by 15%–25% compared to a traditional bioreactor. Conventional bioreactors do not have a liquid flow guide and the temperature is controlled by a jacket. The order of the stirrers is chosen according to the characteristics of the different stirrers. The Six straight-bladed stirrers and Six curved blade stirrers are efficient in breaking up air bubbles and are therefore installed below the air distributor. Liquid flow guides reduce the vortex of the liquid. Larger coolers are beneficial for reducing temperature stability in the bioreactor. The combination of the stirring paddle and air distributor is also inefficient as shown in Figure 3. After calculations, we show that k_L_a = 367.57 h^-1^ for mechanically stirred equipment, which is not conducive to oxygen transfer, and k_L_a = 875.64 h^-1^ for oxygen-enhanced equipment, which promotes oxygen transfer. In addition, the aerated fermentation unit is simple in structure and easy to operate, which is conducive to improving the efficiency of amino acid fermentation, energy savings, and cost savings.

### 3.2 Equipment performance of oxygen-enhanced bioreactors

*C. glutamicum* LS260 is an L-lysine-producing bacterial strain that reaches a high density in the production process. Owing to the performance limitations of conventional equipment, the fermentation process often suffers from insufficient dissolved oxygen levels, which, to some extent, diminishes the advantages of the L-lysine metabolic pathway (Heinemann et al., 1970). Therefore, we designed and applied a new multi-paddle-linked oxygen-enhanced bioreactor. First, we compared the biomass and yield trends in the two bioreactors. As shown in Figure 3A, the growth rate of cells in the pre-fermentation period was similar in terms of biomass. When fermentation proceeded to 10 h, significant differences were observed between the two sets of experiments. In the oxygen-enhanced bioreactor, the biomass OD600 reached 107.2, which was 3.9% higher than that in a conventional bioreactor. The cell growth trends were similar in both experimental groups. Their logarithmic growth period ended after 14 h. The oxygen-enhanced bioreactor provided higher and more stable pairs of dissolved oxygen, which provided the material basis for intracellular energy metabolism. This enabled the cells in group B to better maintain growth viability, and L-lysine production capacity was also enhanced. At 16–36 h of fermentation, the L-lysine productivity was 1.63 g/L/h in the conventional bioreactor and 1.86 g/L/h in the oxygen-enhanced bioreactor, an improvement of 14%. Fermentation proceeded until the end of 36 h, and the final titer of L-lysine in the oxygen-enhanced bioreactor was 185.3 g/L compared to 167.0 g/L in the conventional bioreactor, an increase of 11.0%. In oxygen-enhanced bioreactors, the energy supply within the producer is more adequate and the activity of enzymes related to L-lysine synthesis is higher than that in conventional bioreactors. We further compared the conversion rate between glucose and L-lysine in the two bioreactors, which was 68.56% in the conventional unit and 74.57% in the oxygen-enhanced unit, an increase of 6.01%. This shows that high levels of dissolved oxygen can significantly improve lysine production performance and demonstrates the beneficial effects of oxygen-enhanced equipment.

**FIGURE 4:**
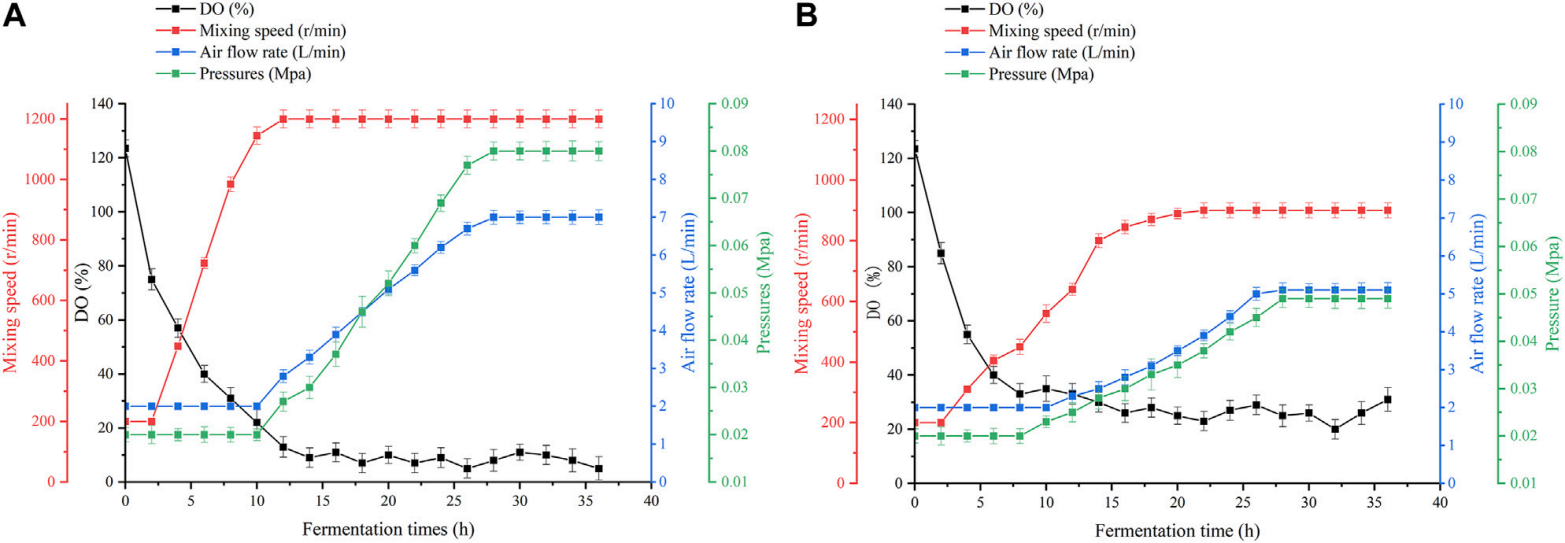
Dissolved oxygen parameters in the two fermentation plants. **(A)** Dissolved oxygen parameters in a conventional bioreactor. **(B)** Dissolved oxygen parameters in an oxygen-enhanced bioreactor.

As shown in Figure 4, in conventional bioreactors, the dissolved oxygen supply is insufficient for 12 h during the fermentation process. After 12 h of fermentation, increasing the speed or aeration of the equipment can increase the dissolved oxygen level for a short time, but it quickly drops to 0%–5%. Even though the maximum speed and aeration of the equipment have been reached, it still cannot significantly raise the dissolved oxygen level. Excessive stirring speed and aeration lead to large amounts of foam and high fermenter pressure, which greatly increases the difficulty of fermentation control, while the stirring shaft of the equipment heats up significantly, thus leading to a different fermentation microenvironment in the bioreactor (Sun et al., 2022). The high-speed rotation of the stirring paddle generates high temperatures. The temperature will rise back to about 65°C. A small number of bacteria that splash onto the stirring shaft because of stirring will die. This is not good for fermentation. In oxygen-enhanced bioreactors, it can be seen that, during the same phase of fermentation, the dependence of the strain on the stirring speed and aeration of the equipment is reduced. We can maintain a stable dissolved oxygen level of approximately 25% by adjusting the speed and airflow. At this level of dissolved oxygen, the strain showed superior lysine synthesis and significantly higher productivity. Interestingly, we found that the difference in productivity owing to high dissolved oxygen decreased as fermentation progressed, as shown in Figure 3B. Therefore, we speculate that, under high dissolved oxygen conditions, the gas-liquid transfer efficiency controlled by the gas film does not affect the ability of the strain to produce acid (Karimi et al., 2021), but rather its ability to take up oxygen. Fortunately, previous studies have shown that oxygen vectors can enhance the uptake of oxygen by microorganisms (Rols and Goma, 1989), thereby enhancing the growth and metabolism of the strain, and further increasing the production of secondary metabolites (You et al., 2021).

### 3.3 Metabolic flow analysis of strain LS260 in two bioreactors

**FIGURE 5:**
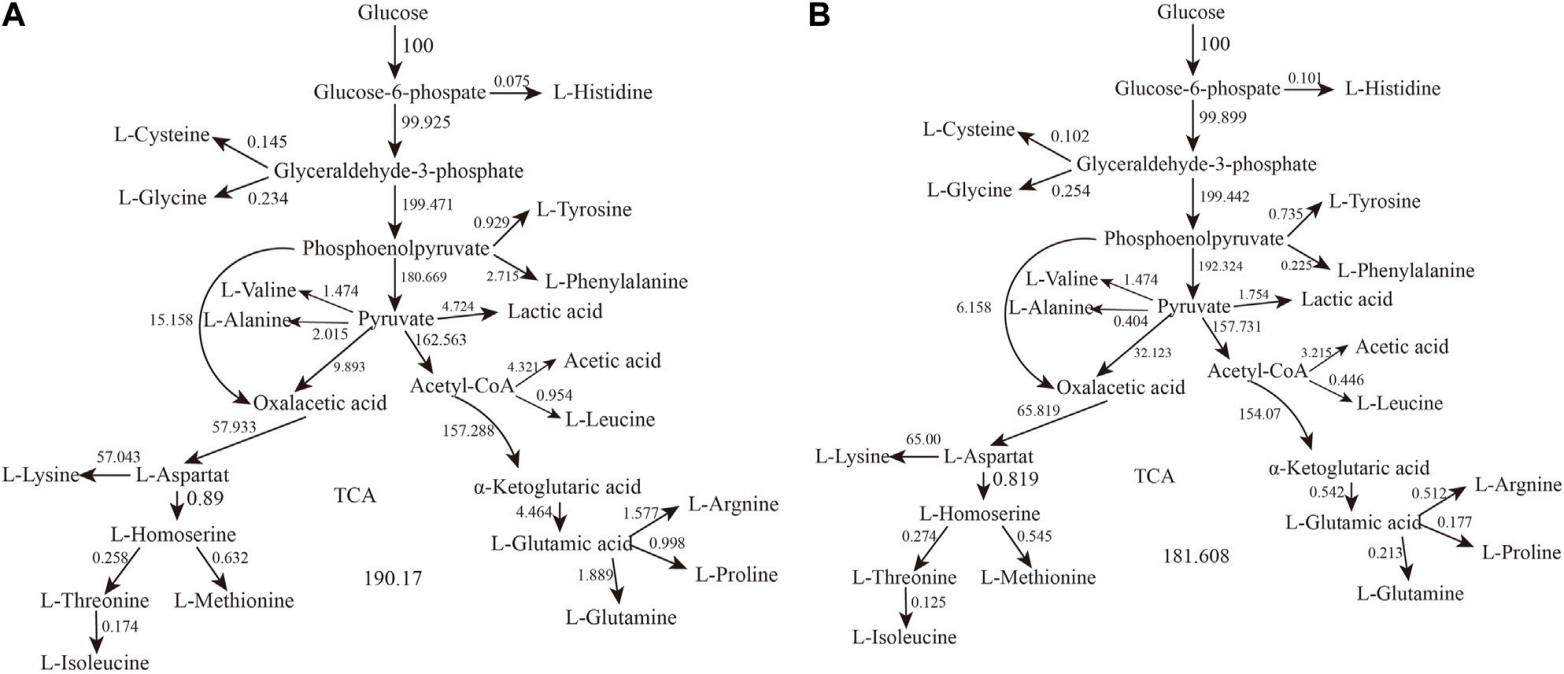
Metabolic network calculation results. **(A)** Metabolic flow in conventional bioreactors. **(B)** Metabolic flow in oxygenated bioreactors.

We analyzed the metabolic flow of L-lysine from strain LS260 in different devices, taking samples with a fermentation time of 16 h for comparison, and found that cellular metabolic processes changed considerably. As shown in Figure 5, the change began with the glycolytic pathway and affected the overall growth trend of the cells. The transient accumulation of aspartic acid in cells with oxaloacetate as a precursor increased by 13.61%, and the entire aspartic acid family metabolic pathway was significantly enhanced, which resulted in an increased metabolic flow to lysine. We compared the metabolic flow of each key node of strain LS260, and in the new device, the pyruvate-OAA node was enhanced. This enhanced the availability of oxaloacetate, which is beneficial for the biosynthesis of the aspartic acid family of amino acids (Yokota et al., 2017; Zhang et al., 2018), and shows promise for application in the synthesis of amino acids in other metabolic pathways. For example, the instantaneous production of amino acids from pyruvate or phosphoenolpyruvate as precursors decreased, with tyrosine down 20.9% and phenylalanine down 91.7%. Leucine declined by 53.2%. At the same time, the l-glutamate synthesis pathway decreased by 87.9%. We believe that there are three reasons for the above phenomenon. (1) The increase in k_L_a of the equipment enhanced bacteriophage metabolism, which is the material and energy basis for bacteriophage growth and lysine production (Inui et al., 2007). (2) The synthesis of other amino acids and organic acids decreased because the enzymatic activity of the lysine synthesis pathway was higher than that of other metabolic pathways (Barksdale, 1970), and the limited oxygen availability and oxygen uptake capacity caused the metabolic flow of C to be more in the direction of lysine synthesis. This means that lysine production and conversion rates increased (3) because the pyruvate-oxaloacetate metabolic node fixed CO_2_ and the enhanced metabolic flux from this node reduced some of the CO_2_ released from the TCA cycle, which also helped increase the conversion rate between glucose and lysine.

### 3.4 Selection of the oxygen vectors

In the above-mentioned study, we found that high levels of dissolved oxygen promoted lysine production by strain LS260. Oxygenated bioreactors had a 2.38-fold increase in oxygen supply capacity, but the cellular uptake of oxygen was limited by gas-liquid transfer controlled by the gas membrane. We attempted to improve oxygen transfer by adding 1% oxygen vectors to the fermentation culture system. To determine the type of oxygen carrier, three oxygen vectors were selected, n-dodecane, perfluorocarbon, and liquid paraffin, and their effects on the fermentation parameters of strain LS260 were investigated.

**FIGURE 6:**
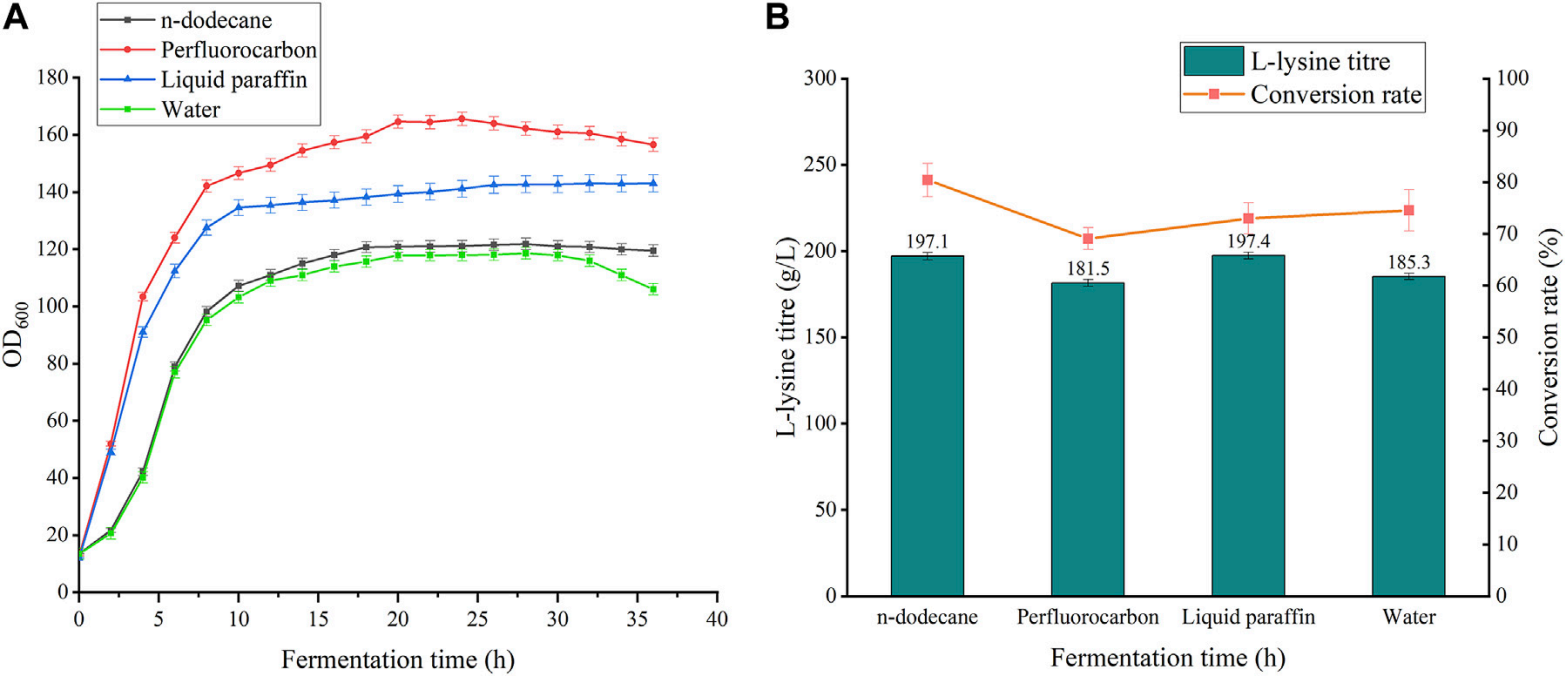
Effects of different oxygen vectors on the fermentation process of strain LS260. **(A)** Cell growth curves of different oxygen vectors. **(B)** Lysine titers and glucose-lysine conversion of different oxygen vectors.

As shown in Figure 6, the addition of all three oxygen vectors had a boosting effect on bacterial growth compared to the 5 L batch replenishment fermentation without oxygen vectors. Perfluorocarbon had the most significant boosting effect on the biomass of the bacteria, with a 39.6% increase, but with a significant decline in the late stages of the fermentation. However, the uncontrolled growth of bacteria resulted in a decrease in lysine production to 181.5 g/L and a 5.47% decrease in conversion. The growth curve of the fermentation process with the addition of n-dodecane and liquid paraffin was relatively smooth with nonsignificant periods of decline. The addition of n-dodecane increased fermentation biomass by 2.78%, lysine yield by 6.53%, and conversion by 5.83%. The experimental group with the addition of liquid paraffin had a 21.22% increase in biomass, a 6.53% increase in yield, and a 1.57% decrease in conversion rate. Oxygen vectors can promote the growth of bacteria, but excessive bacterial density leads to a disruption of metabolic flow, which in turn affects the ability to synthesize glucose for lysine conversion. N-dodecane is more suitable than the other two oxygen vectors for the fermentation of lysine by strain LS260.

### 3.5 Effects of n-dodecane addition at different stages on L-lysine production by strain LS260

The oxygen demand of bacteria varies considerably at different stages of growth, and it is particularly important to choose the appropriate time and method of addition of n-dodecane. We varied the time of fermentation addition according to the stage of bacterial growth, adding oxygen vectors at 0, 8, 16, and 24 h.

**FIGURE 7:**
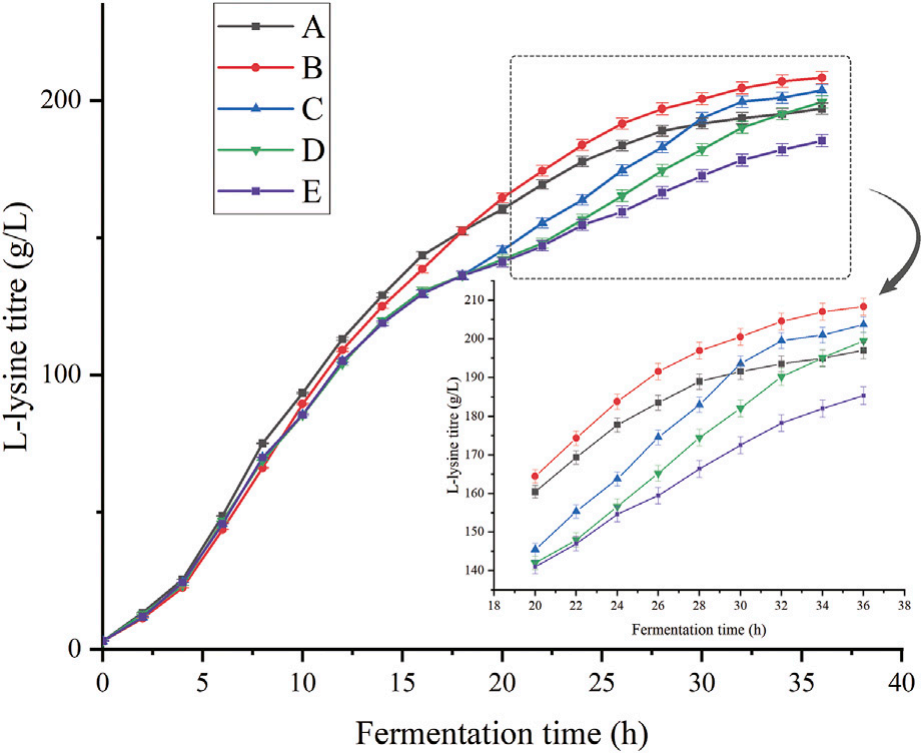
Effects of different addition times of oxygen vectors on the production of L-lysine. **(A)** Addition of n-dodecane from the start of fermentation. **(B)** Addition of n-dodecane at 8 h of fermentation. **(C)** Addition of n-dodecane at 16 h of fermentation. **(D)** Addition of n-dodecane at 24 h of fermentation **(E)** No n-dodecane was added to the fermentation broth.

As shown in Figure 7, the addition of oxygen vectors at all times was beneficial for lysine fermentation. The addition of oxygen vectors at 8 h of fermentation was more effective than that at other times. Compared to the fermentation culture without the addition of oxygen vectors, the batch replenishment fermentation yields at 0, 8, 16, and 24 h increased by 6.31%, 12.44%, 9.93%, and 7.39%, respectively. The addition of n-dodecane at the beginning of fermentation increased the intensity of production in the early stages of fermentation and caused the premature death of bacteria to some extent in the later stages. The best results were achieved by adding the oxygen vectors at the 8th hour of fermentation, where the yield reached 208.36 g/L and the conversion rate reached 83.3%. According to the DO graph shown in Figure 6 in 3.2, at approximately 10 h of fermentation, the dissolved oxygen already dropped to approximately 20% and showed a trend of hypoxia. Addition at 8 h of fermentation increased the oxygen uptake capacity of cells before the onset of hypoxia, eliminating the reduced productivity and cellular damage associated with it. Therefore, the addition of oxygen vectors at 16 h and 24 h into the fermentation process, although boosting the bacterium’s ability to produce lysine, does not fully offset the damage caused by the lack of oxygen. During the course of the study, it was found that the glucose supplementation was initiated when its concentration decreased below 5 g/L (Addition of 50 g/L of glucose monohydrate to the fermentation medium from the beginning.), and the rate of glucose consumption differed between the fermentations with and without n-dodecane. L-lysine fermentation with n-dodecane required glucose supplementation from 3.3 h, whereas non-n-dodecane fermentation required glucose supplementation from 5.2 h. In the absence of glucose, the aerobic carriers continued to grow for approximately 1.5 h and the anaerobic carriers stopped growing immediately. We speculate that there may be metabolic pathways in *C. glutamicum* that use long-chain alkanes as a source of carbon and energy to provide the organic carbon required for growth. Therefore, maintaining glucose concentration in the fermentation system plays an important role in ensuring the concentration of hydrocarbons in the system.

In addition, industrial production of L-lysine requires the addition of large quantities of substances, such as corn pulp. These nutritional additives contain large amounts of natural organic substances, such as proteins, sugars, and peptides. During the fermentation process, the viscosity of the fermentation broth increases owing to high-speed agitation and extensive aeration, coupled with the growth of biomass, making it extremely easy to produce large amounts of foam. Due to the oily substance n-dodecane, fermentation produces very little foam because of its hydrophobic properties. Its presence changes the surface tension of the bubbles, and therefore foam can be eliminated without the addition of a defoamer.

## 4 Conclusion

In this study, we designed and developed an aerobic bioreactor. This bioreactor optimizes the aeration mix by using internal deflectors and multistage propellers. The bioreactor k_L_a increased from 367.57 to 875.64 h^-1^, an improvement of 238.22%. The results show that the oxygen supply capacity of the oxygen-enhanced bioreactor was better than that of a conventional bioreactor. Dissolved oxygen increased by 20% during fermentation’s middle and late stages. Strain LS260 grew with increased mid-to late-stage viability, yielding 185.3 g/L of L-lysine and 74.57% conversion, an increase of 11.0% and 6.01%, respectively, over the conventional bioreactor, and an 8.2% increase in productivity of 2.57 g/L/h. To further enhance L-lysine production and sugar-acid conversion, we investigated the effects of oxygen carriers on L-lysine production during batch replenishment fermentation with *C. glutamicum*. A comparison of the effects of the three oxygen vectors on the production of L-lysine from LS260 fermentation revealed that the oxygen vector promoted cell growth. N-dodecane was the most suitable of the three oxygen carriers, and the growth of the cell was smoother under these conditions. The result was a 38.2% increase in biomass, an increase in L-lysine production to 227.1 g/L, and a sugar-acid conversion rate of 80.4%. Next, the effects of different n-dodecane addition times on the final yield and conversion were investigated. Compared with the fermentation culture without the addition of oxygen carriers, the experimental group increased the yield by 6.31%, 12.44%, 9.93%, and 7.39% with the addition of n-dodecane at 0, 8, 16, and 24 h, respectively. The conversion of glucose to lysine was increased by 5.83%, 8.73%, 7.13%, and 6.13%, respectively. The best results were achieved by adding oxygen carriers at the 8th hour of fermentation, at which point the fermentation yield reached 208.36 g/L and the conversion rate reached 83.3%. In addition, an incidental discovery in this study was that n-dodecane may be utilized as a carbon source when the bacterium lacks it. Foam production during fermentation is significantly reduced by the presence of n-dodecane, which is beneficial for fermentation control and equipment.

This newly oxygenated transformer can be applied in the fermentation process with high bacterial density, which can effectively improve the problem of insufficient oxygen supply in the fermentation process and has great application prospects, which is a reference for the bioengineering industry. Based on the physical properties of n-dodecane, n-dodecane is relatively safe for application in fermentation processes. Because n-dodecane is insoluble in water and less dense than water, it can be easily separated from the fermentation broth and recycled. The low price of n-dodecane and recycling, which has little impact on costs, can effectively improve the economic efficiency of high-value-added and high-yield products. At the same time, we are the first to identify the use of long-chain alkanes for growth in *C. glutamicum*, which has significant implications for subsequent carbon source replacement studies.

## Data Availability

The original contributions presented in the study are included in the article/supplementary material, further inquiries can be directed to the corresponding author.
